# SC35 promotes splicing of the C5-V6-C6 isoform of CD44 pre-mRNA

**DOI:** 10.3892/or.2013.2812

**Published:** 2013-10-24

**Authors:** TIING JEN LOH, HEEGYUM MOON, SUNGHEE CHO, DA-WOON JUNG, SEONG-EUI HONG, DO HAN KIM, MICHAEL R. GREEN, XUEXIU ZHENG, JIANHUA ZHOU, HAIHONG SHEN

**Affiliations:** 1 School of Life Sciences, Gwangju Institute of Science and Technology, Gwangju 500-712, Republic of Korea; 2 Howard Hughes Medical Institute and Program in Gene Function and Expression, University of Massachusetts Medical School, Worcester, MA, USA; 3 Jiangsu Key Laboratory of Neuroregeneration, Nantong University, Nantong, P.R. China

**Keywords:** CD44, cancer, pre-mRNA splicing, SC35, V6 exon

## Abstract

CD44 is a cell membrane glycoprotein that mediates the response of cells to their cellular microenvironment and regulates growth, survival, differentiation and motility. CD44 pre-mRNA contains 20 exons, 10 of which are alternatively spliced. Among the CD44 spliced variants, one of the V6 exon-containing isoforms, the V4–7 variant which contains variable exons 4, 5, 6 and 7, confers metastatic potential to non-metastatic cells. However, the splicing regulation of the V6 exon is not completely understood. SC35 is an arginine-serine rich protein that regulates alternative splicing of various pre-mRNAs. In the present study, we established a stable cell line which indicates inclusion or skipping of the V6 exon with the RFP or GFP signal. Using this stable cell line, we found that the V6 exon and flanking introns of CD44 pre-mRNA contained SC35 response elements that regulate V6 splicing. RT-PCR analyses of the endogenous CD44 splicing showed that SC35 promotes the production of the C5-V6-C6 isoform. shRNA knockdown of SC35 showed that reduced expression of SC35 decreased expression of the V6 exon-containing isoforms. Our results reveal a novel mechanism of CD44V6 splicing.

## Introduction

CD44 is a cell membrane glycoprotein, which mediates the response of cells to their cellular microenvironment and regulates growth, survival, differentiation and motility (1–3). The function of CD44 depends on its ligands. Hyaluronic acid mediates the tumor-suppressor function of CD44, while growth factors regulate the growth promotion function of CD44 (4). CD44 is encoded by a single gene consisting of 20 exons. Exons 1–5 and exons 16–20 are constitutively spliced, and are included in all of the CD44 mRNA isoforms. Exons 6–16 (V exons) are differently included or skipped to generate a large variety of splicing variants (5). The amino terminal domain of the standard isoform is separated from the plasma membrane by an extracellular, membrane-proximal stem structure of CD44 protein. The stem structure can be different due to the alternative splicing of stem-encoding variant exons (1,6).

Among the various CD44 isoforms, the V6 exon-containing isoforms (CD44V6) have been implicated in tumorigenesis (6), tumor cell invasion and metastasis (7,8). It was shown that CD44V4-V7 conferred metastatic potential to cells of a non-metastatic rat tumor cell line (7). Immunohistochemistry analysis demonstrated a much higher expression of CD44V6 in various types of tumors when compared with that in normal tissues (9–11). Due to its significantly high expression, CD44V6 antibody-based cancer therapy was developed (12,13). The CD44V6-containing isoform forms a complex with the extracellular hepatocyte growth factor (HGF) and its tyrosine kinase receptor Met (14,15). Formation of this complex (CD44V6-HGF-Met) activates Met-dependent Ras signaling (14,16) through the association of ERM (ezrin-radixin-moesin) (17–19) to the cytoplasmic tail of CD44. However, the splicing mechanism of CD44V6 is not yet clear.

Pre-mRNA splicing is essential for gene expression in higher eukaryotes (20). Alternative splicing produces diverse proteins from a gene. Regulation of alternative splicing plays key roles in signal transduction and development. Deregulation of alternative splicing causes various types of diseases including cancer (21–24). Pre-mRNA splicing requires critical sequences on pre-mRNA called splicing signals, which include the 5′ splice site, the 3′ splice site, the polypyrimidine tract (PPT) and branch point (25,26). Pre-mRNA splicing is regulated by *cis*-acting elements and *trans*-acting elements (27–29). *Cis*-acting elements are also called splicing enhancers or inhibitors which are specific RNA sequences located at exons or introns. *Trans*-acting elements are proteins which promote exon inclusion or skipping.

SC35 is an SR (serine-arginine rich) protein that includes RRMs (RNA recognition motifs) and RS (arginine-serine rich) domain (30). SR proteins participate in multiple steps of splicing including U1 snRNP binding to the 5′ splice site and U2 snRNP binding to the branch point. The RS domain of SR proteins functions as an activator, whereas RRMs provide the binding sites for RNA (31–33). SR proteins also play additional roles in transcription, RNA stability, mRNA transport and mRNA translation (34). SC35 plays key roles in constitutive and alternative splicing in higher eukaryotes.

In the present study, we created a stable cell line which reports V6 exon skipping and inclusion of CD44 pre-mRNA with green fluorescence protein (GFP) or red fluorescence protein (RFP), independently. With this cell line, we identified that the V6 exon and flanking introns contain SC35 responsive elements. Furthermore, we found that overexpression of SC35 promoted C5-V6-C6 isoform production of CD44; knockdown of SC35 reduced CD44V6 expression.

## Materials and methods

### Construction of pFlare-v6 mini-gene

A reporter construct was generated using standard cloning techniques. The CD44 genomic DNA, which includes the v6 exon and flanking introns (500 bp each), was PCR amplified from the Human Genomic DNA Library (Promega, Madison, WI, USA). The PCR product was cloned in *Mlu*I and *Bam*HI enzyme sites in pFlare9A-Dup34 which was provided by Dr D.L. Black from the Howard Hughes Medical Institute (Worcester, MA, USA) (35).

### Cell culture and generation of the stable cell line

Breast cancer cell line, MCF-7, was obtained from ATCC and maintained in Dulbecco’s modified Eagle’s medium (DMEM) supplemented with 10% fetal bovine serum (FBS) at 37°C in a humidified 5% CO_2_ atmosphere. Plasmid transfection into MCF-7 cells was carried out with polyethyleneimide (PEI) according to the manufacturer’s instruction. PEI (2 μg) was mixed with 1 μg of the pFlare-v6 plasmid in 100 μl of DMEM and incubated for 10 min. The mixture was applied to cells in 900 μl of DMEM supplemented with FBS. Four hours later, the medium was replaced. The transfected cells were selected under 1 mg/ml G418 (Sigma) for 3 weeks. Stably transfected clones were validated by RT-PCR and fluorescence microscopy.

### RT-PCR

Total RNA was extracted from transfected MCF-7 cells using RiboEx reagent (GeneAll) following the manufacturer’s protocol. Total RNA (1 μg) was reverse transcribed using oligo(dT) primer using ImProm-II™ reverse transcriptase (Promega) following the manufacturer’s protocol. The cDNA (1 μl) was amplified by PCR using G-Taq polymerase (Cosmo Genetech Co., Ltd., Seoul, Korea). The primers used are as following: pFlarev6 forward (5′-GGA AGA GTT GGT GGT GAG G-3′) and pFlarev6 reverse (5′-GGT GCA GAT GAA CTT CAG G-3′); v6 forward (5′-TCC AGG CAA CTC CTA GTA GT-3′) and v6 reverse (5′-CAG CTG TCC CTG TTG TCG AA-3′); GAPDH forward (5′-ACC ACA GTC CAT GCC ATC A-3′) and GAPDH reverse (5′-TCC ACC ACC CTG TTG CTG TA-3′). For the endogenous CD44, RT-PCR was conducted as previously described (36). A specific primer, CD44RT (5′-ATG CAA ACT GCA AGA ATC-3′) was used for reverse transcription. PCR was carried out with C5v6 forward (5′-ATC CCT GCT ACC ATC CAG GCA AC-3′) and CD44E7 reverse (5′-TTT GCT CCA CCT TCT TGA CTC C-3′).

### Fluorescence microscopy

Fluorescence analyses were performed using an Olympus IX71 inverted microscope and a ×20 LCPlanF1 Ph1 objective. Images were composed using MetaMorph software and saved as JPEG files.

### shRNA treatment

The shRNA lentivirus was generated by co-transfection of the pLKO.1 plasmid encoding the SC35 mRNA matching sequence or the non-silencing sequence (Open Biosystems) and PSPAX2 and PMD2G helper plasmids into 293T cells using polyethyleneimide (PEI). The medium was replaced after 24 h and incubated for another 24 h. The supernatants containing the lentiviruses were harvested with a 0.45-μm filter. MCF-7 cells were seeded in a 6-well plate one day prior to infection. The lentiviruses containing the supernatants were added to the cells supplemented with 8 μg/ml Polybrene. After a 72-h infection, RNAs were extracted for RT-PCR.

## Results

### A stable cell line reports V6 exon splicing of CD44 pre-mRNA

**Figure 1:**
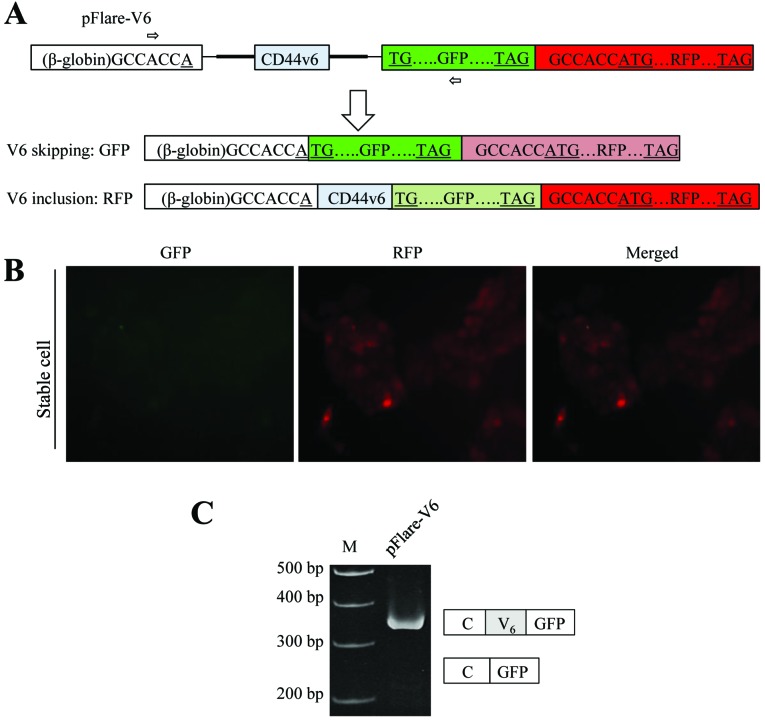
A stable cell line indicates V6 exon splicing of CD44 by expression of green fluorescence protein (GFP) or red fluorescence protein (RFP). (A) Strategy of pFlare-V6 mini-gene constructs. The V6 exon of CD44 is shown in the middle; flanking introns (500 nt each) are shown with thick lines. The introns other than CD44 are shown as thin lines. V6 exon skipping of CD44 induces GFP expression; V6 exon inclusion induces RFP expression. The GFP signal is shown as green when it is expressed; the RFP signal is shown as red when it is expressed. Initiation codons and stop codons are underlined. The primers used for RT-PCR are shown as arrows. (B) Fluorescence microscopy of the stable cell line which expresses pFlare-V6. (C) RT-PCR analysis of the pFlare-V6 stable cell line. The V6 exon included product is C-V6-GFP; the V6 exon skipped product is C-GFP.

In order to identify the regulatory factors for V6 exon splicing of CD44 pre-mRNA, we constructed a mini-gene with the pFlare-RFP/GFP reporter system. It was previously shown that the start codon of GFP is split into a constant β-globin exon and GFP exon (35). V6 exon and flanking introns of CD44 were inserted between the constitute exon and GFP exon (Fig. 1A). GFP is expressed when the flanking test exon (V6 exon of CD44) is skipped, whereas another start codon on RFP is out of frame in the pFlare-V6 plasmid. If V6 exon of CD44 is included, the start codon for GFP expression is abolished. Then another start codon which is located at RFP will be used for translation of RFP (Fig. 1A). The pFlare-V6 plasmid was transfected into MCF-7 cells, and the stable cell line (pFlare-V6-MCF-7) was established by G418 selection for three weeks. The results in Fig. 1B show that the red fluorescence protein (RFP) was highly expressed whereas green fluorescence protein (GFP) was almost not detected in the stable cell line. Therefore, we hypothesized that the CD44V6 included form should be the dominant isoform, and that the skipped form is the minimum isoform for the pFlare-V6 stable cell line. In order to test this possibility, we performed RT (reverse-transcriptase)-PCR analysis. RNA was extracted from the stable cell line. Primer sets that base pair with constant exon and RFP separately were used for RT-PCR analysis (Fig. 1A). The results in Fig. 1C show that the CD44V6 included isoform was dominantly expressed from the stable cell line; whereas the CD44V6 skipped isoform was not detectable at a significant level. Thus, the expression of RFP and GFP indicates V6 exon splicing. Therefore, the stable cell line expressing pFlare-V6 was able to be applied for the identification of factors which regulate V6 exon splicing of CD44 through targeting V6 exon and flanking introns.

### V6 exon and flanking introns of CD44 contain SC35 responsive elements

**Figure 2:**
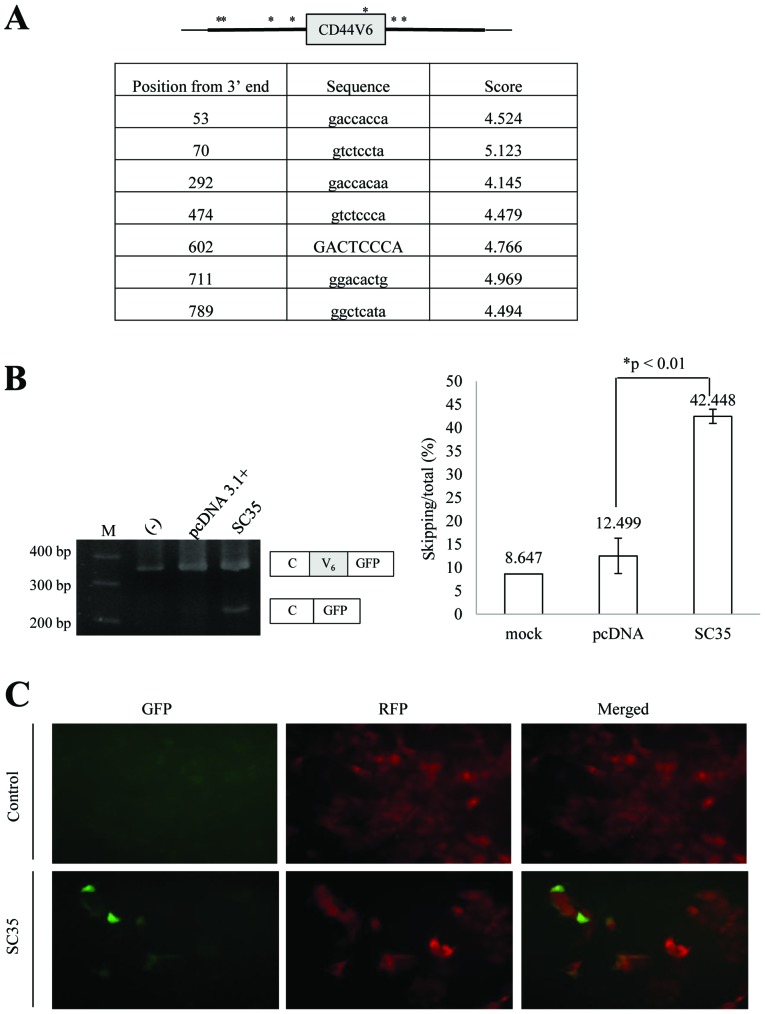
V6 exon and flanking introns of CD44 contain SC35 response elements. (A) Potential SC35 binding sites on CD44 exon V6 and flanking introns with scores. (B) RT-PCR analysis of V6 exon splicing of CD44 from pFlare-V6-expressing stable cell lines which were transected with the SC35 or control (pcDNA3.1+) plasmid. Quantification of the results was carried out using ImageJ and analysis was performed using t-test with n=3; P<0.01. (C) Fluorescence microscopic analysis of the pFlare-V6 stable cells that were transfected with the pcDNA3.1+ or SC35 plasmid.

In order to test the possibility that SC35 regulates CD44V6 splicing, we applied a bioinformatics approach. Using a bioinformatics tool (ESE finder) (37), we found that there is one potential SC35 binding site on the V6 exon, four potential binding sites on the upstream introns and two potential binding sites on the downstream introns, which are located at 53, 70, 292, 474, 602, 711 and 789 bp downstream from the 3′ end of the inserted V6 exon and flanking introns (Fig. 2A). We hypothesized that SC35 regulates CD44V6 splicing. To test this possibility, we expressed SC35 in the pFlare-V6-expressing cells. RNA was extracted; RT-PCR analysis was performed for the splicing of the V6 exon. As expected, the results in Fig. 2B show that SC35 significantly promoted-skipping of the V6 exon (by ~34%). By contrast, expression of the control plasmid (pcDNA3.1+) did not cause a significant change in V6 exon splicing. This result was consistent with the fluorescent expression of stable cells as shown in Fig. 2C. Thus, we concluded that CD44V6 and flanking introns contain SC35 response elements.

### SC35 promotes the production of the endogenous C5-V6-C6 isoform of CD44

**Figure 3:**
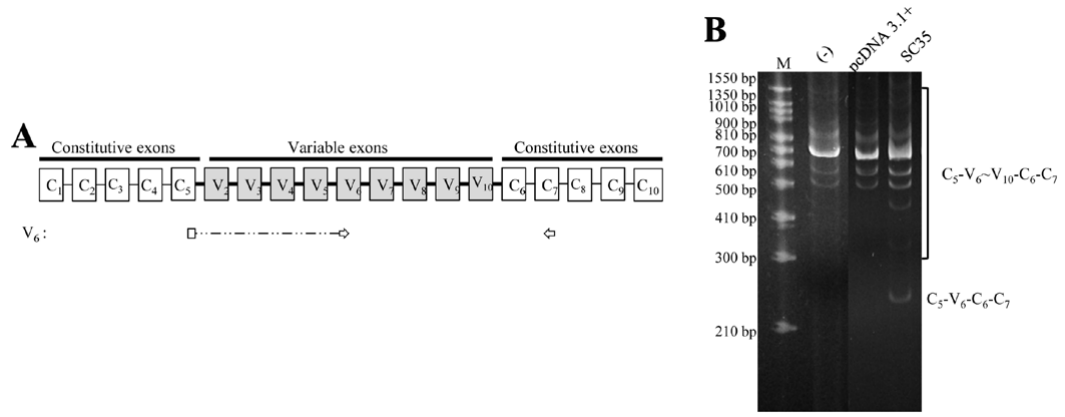
SC35 overexpression promotes production of the endogenous C5-V6-C6 isoform of the CD44 gene. (A) Primers for detecting endogenous CD44V6 are shown as arrows. (B) RT-PCR analysis of endogenous CD44V6 with RNAs extracted from MCF-7 cells which overexpress SC35 or control plasmid.

We next aimed to ascertain whether SC35 regulates the splicing of the endogenous V6 exon. To detect V6-containing isoforms, we used primer sets that are base paired with junction C5V6 and C7 separately (Fig. 3A). The isoforms detected also included V7, V8, V9 or V10 exons. The MCF-7 cell line is a non-metastatic human breast cancer cell line. The results in Fig. 3B showed that the MCF-7 cell line does not express the C5-V6-C6 isoform of CD44, in which only V6 is included but other various exons are excluded. Expression of SC35 promotes the production of the C5-V6-C6 isoform. The production of other V6 exon-containing isoforms, which includes other various isoforms in addition to V6 (C5-V6~V10-C6), was not altered upon SC35 expression. Therefore, we concluded that SC35 promotes the production of the C5-V6-C6 isoform.

### Knockdown of SC35 reduces endogenous CD44V6 expression

**Figure 4:**
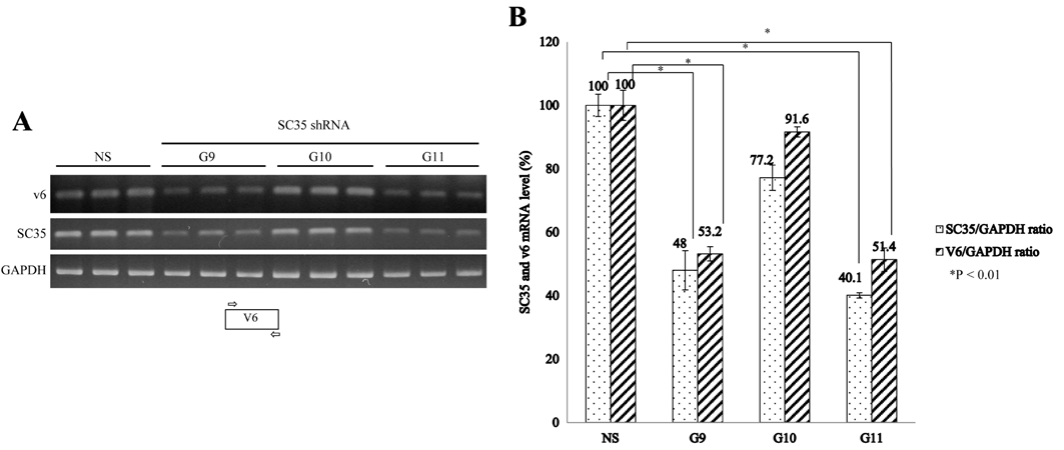
Knockdown of SC35 reduces the expression of the CD44V6 isoform. (A) RT-PCR results of the CD44V6 isoform in cells treated with non-silencing (NS) shRNA, and three different shRNAs targeting SC35 (G9, G10 and G11). All experiments were conducted in triplicate. Primers are shown as arrows. GAPDH was used as a control. (B) Quantification of the results of RT-PCR analysis is described in A using ImageJ and t-test was performed; n=3. The results were normalized to the ratios of SC35/GAPDH and V6/GAPDH. P<0.01.

We next aimed to ascertain whether SC35 knockdown affects V6 exon splicing of CD44. We applied three types of shRNAs (G9, G10 and G11) to knockdown SC35 mRNA. Fig. 3A shows that G9 and G11 shRNAs significantly reduced the expression of SC35 as shown with RT-PCR with SC35-specific primer sets. We found that G10 shRNA which was designed to target SC35 and non-silencing shRNA did not reduce SC35 expression. To test the effects carefully, we performed triplicate experiments for every shRNA. RNA was extracted from the cells treated with shRNA virus and untreated cells; RT-PCR was performed with primer sets that were base paired with the V6 exon (Fig. 4A). Thus, the PCR products represented all of the V6-containing isoforms (CD44V6). The RT-PCR results were normalized to the ratio of SC35/GAPDH and V6/GAPDH (Fig. 4B). The results in Fig. 4A show that V6 expression was significantly reduced after infection with the G9 and G11 shRNA virus. In contrast, V6 expression was not significantly altered upon G10 and non-silencing shRNA virus infection. Therefore, we concluded that knockdown of SC35 reduces expression of the CD44V6 isoforms. The quantitation of results is shown in Fig. 4B. G9 and G11 shRNAs reduced the V6 expression by ~48 and ~49% as shown by the average of the V6/GAPDH ratio. However, G10 shRNA did not induce a significant decrease in V6 exon expression. V6 expression was reduced after G9 and G11 shRNA infection as shown by the V6/GAPDH ratio, whereas G10 shRNA infection did not induce the alteration of V6 expression significantly. Therefore, we concluded that knockdown of SC35 induces a decrease in CD44V6 expression.

## Discussion

SR proteins play important roles in constitutive and alternative splicing (38–40). SC35 has been reported to play an active role in transcriptional elongation (41) and mRNA stability (42). In the present study, we performed a systematic RT-PCR analysis to determine the role of SC35 in CD44 pre-mRNA splicing. In the present study, we created a stable cell line that expressed the V6 exon of CD44 and its flanking introns. Expression of RFP (red florescence protein) indicated inclusion of the V6 exon, whereas (green florescence protein) GFP expression indicated V6 exon skipping. With this stable cell line, we found that SC35 promotes V6 exon skipping in the mini-gene. These results indicate that V6 exon and flanking introns of CD44 pre-mRNA contain SC35 response elements. RT-PCR analysis with endogenous CD44 pre-mRNA demonstrated that SC35 promotes the C5-V6-C6 expression of the CD44 pre-mRNA. By contrast, knockdown of SC35 with shRNA reduced expression of the V6-containing isoform. Collectively, our results indicate that SC35 promotes CD44V6 inclusion.

### V6 exon and flanking introns of CD44 pre-mRNA contain response elements for SC35

Our strategy for constructing a pFlare-V6 mini-gene is that GFP is expressed when the V6 exon of CD44 is skipped. By contrast, RFP is expressed while CD44V6 is included. Our microscopy results demonstrated that the stable cell line expressed only RFP. RT-PCR results showed that the V6 exon of CD44 was dominantly included in the pFlare-V6-transfected MCF cells. Consistent with the results, microscopic analysis demonstrated that the stable cell line expressed only RFP. pFlare plasmid contained the V6 exon and flanking introns (500 nt each) of CD44 pre-mRNA. The first and last exon of the mini-gene (pFlare-V6) was not from the CD44 gene; thus, the mini-gene can be applied for the identification of *trans*-acting response elements on V6 exon and flanking introns. Based on a bioinformatics approach we hypothesized that V6 and flanking introns contain potential SC35-binding sites. As expected, SC35 regulates V6 exon splicing of CD44 in the pFlare-V6 pre-mRNA. Our results demonstrated that CD44V6 and flanking introns contain SC35 response elements.

### SC35 promotes the production of the C5-V6-C6 isoform of CD44

The existence of SC35 response elements on CD44V6 and flanking introns raised the possibility that SC35 regulates endogenous V6 exon splicing of CD44 pre-mRNA. We found that SC35 induced the expression of the C5-V6-C6 isoform of CD44, which was not significantly expressed in the non-metastatic breast cancer cell line MCF-7.

### Knockdown of SC35 reduces the expression of the CD44V6 isoform

We found that SC35 knockdown induced a decrease in CD44V6 expression. Therefore, we conclude that SC35 has a critical function in the regulation of CD44V6 splicing.
